# Human Seroprevalence for Dengue, Ross River, and Barmah Forest viruses in Australia and the Pacific: A systematic review spanning seven decades

**DOI:** 10.1371/journal.pntd.0010314

**Published:** 2022-04-29

**Authors:** Eugene T. Madzokere, Wei Qian, Julie A. Webster, Daniel M. H. Walker, Elisa X. Y. Lim, David Harley, Lara J. Herrero

**Affiliations:** 1 Institute for Glycomics, Griffith University, Gold Coast Campus, Southport, Australia; 2 Centre for Clinical Research, University of Queensland, Brisbane, Australia; 3 QIMR Berghofer Medical Research Institute, Brisbane, Australia; Centers for Disease Control and Prevention, UNITED STATES

## Abstract

**Background:**

Dengue (DENV), Ross River (RRV) and Barmah Forest viruses (BFV) are the most common human arboviral infections in Australia and the Pacific Island Countries and Territories (PICTs) and are associated with debilitating symptoms. All are nationally notifiable in Australia, but routine surveillance is limited to a few locations in the PICTs. Understanding the level of human exposure to these viruses can inform disease management and mitigation strategies. To assess the historic and current seroprevalence of DENV, RRV and BFV in Australia and the PICTs we conducted a systematic literature review of all published quantitative serosurveys.

**Methodology and principal findings:**

The Preferred Reporting of Items for Systematic Reviews and Meta-Analyses procedures were adopted to produce a protocol to systematically search for published studies reporting the seroprevalence of DENV, RRV and BFV in Australia and the PICTs. Data for author, research year, location, study population, serosurvey methods and positive tests were extracted. A total of 41 papers, reporting 78 serosurveys of DENV, RRV and BFV including 62,327 samples met the inclusion criteria for this review. Seroprevalence varied depending on the assay used, strategy of sample collection and location of the study population. Significant differences were observed in reported seropositivity depending on the sample collection strategy with clinically targeted sampling reporting the highest seroprevalence across all three viruses. Non-stratified seroprevalence showed wide ranges in reported positivity with DENV 0.0% – 95.6%, RRV 0.0% – 100.0%, and BFV 0.3% – 12.5%. We discuss some of the causes of variation including serological methods used, selection bias in sample collection including clinical or environmental associations, and location of study site. We consider the extent to which serosurveys reflect the epidemiology of the viruses and provide broad recommendations regarding the conduct and reporting of arbovirus serosurveys.

**Conclusions and significance:**

Human serosurveys provide important information on the extent of human exposure to arboviruses across: (1) time, (2) place, and (3) person (e.g., age, gender, clinical presentation etc). Interpreting results obtained at these scales has the potential to inform us about transmission cycles, improve diagnostic surveillance, and mitigate future outbreaks. Future research should streamline methods and reduce bias to allow a better understanding of the burden of these diseases and the factors associated with seroprevalence. Greater consideration should be given to the interpretation of seroprevalence in studies, and increased rigour applied in linking seroprevalence to transmission dynamics.

## Author summary

Mosquito-borne viruses contribute significantly to the global burden of diseases. Understanding infection rates in human populations is important for informing management of disease burden and public health policy. Serosurveys measure population exposure and immunity after arbovirus infections. We systematically reviewed serosurveys conducted for dengue virus (DENV), Ross River virus (RRV), and Barmah Forest virus (BFV) in Australia and the PICTs. We identified 41 studies reporting 78 serosurveys published between 1966 and 2020. For DENV we found the highest seroprevalence, greater than 95.0%, was reported in American Samoa. For RRV we found early evidence (1960–1969) of circulation outside Australia. There were three studies of BFV reporting 16 serosurveys within Australia and reporting low seroprevalence (<13%). Researchers used different serological methods so study comparisons must be nuanced.

## Introduction

Arboviruses pose a significant health threat to more than four billion people worldwide and are therefore a global public health priority [1]. Efforts to manage the global spread of arboviruses are hampered by asymptomatic infections, limited availability of rapid diagnostics, complicated vector-host dynamics, travel, and environmental change [2,3]. This is evidenced through the globally increasing number and severity of arboviral epidemics, including zika, West Nile, and dengue viruses, over the past 50 years [2,4]. Human serosurveys are an epidemiological tool regularly adopted to measure arboviral transmission in a population. When serosurveys are conducted over wide temporal and spatial scales, they can be used to (1) identify exposure rates and viruses currently circulating within populations, (2) identify susceptible populations in which outbreaks may occur, and (3) distinguish symptomatic from asymptomatic infections. Serosurveys measure population exposure and immunity and this is useful for assessing population risk and building predictive transmission models.

Several human arboviruses circulate in Australia and the Pacific Island Countries and Territories (PICTs), including dengue virus (DENV), Ross River virus (RRV) and Barmah Forest virus (BFV). Of these, DENV is associated with the greatest public health burden globally. Dengue is a single stranded, positive-sense RNA flavivirus mainly transmitted via human-mosquito-human transmission by *Aedes aegypti* (Linnaeus) and *Aedes albopictus* (Skuse) mosquitoes [5]. DENV comprises four serotypes (DENV-1, DENV-2, DENV-3 and DENV-4) which have 65% genomic similarities and near identical clinical syndromes with all serotypes occupying the same ecological niches [6]. Regional variation in epidemiology arises because of host (e.g. behaviour, population age structure), agent (e.g. co-circulation of serotypes, extrinsic incubation period) and environmental (e.g. vector behaviour, built environment, global climate change) factors [7]. Infection ranges from asymptomatic to classic dengue fever and more severe forms of disease, such as dengue haemorrhagic fever and dengue shock syndrome [8]. Severe DENV cases are associated with high human morbidity and around 20,000 deaths per year globally [9]. Within Australia, managing DENV disease is estimated to cost AU$17 million annually [10]. Recently, there has been an increase in number of DENV cases reported in some of the PICTs, including the Solomon Islands, Vanuatu, Fiji and Palau [11].

Both RRV and BFV are zoonotic, single stranded, positive-sensed RNA alphaviruses maintained by multiple vector and host species throughout Australia [12,13]. RRV is the most common and widespread arbovirus in Australia, accounting for more than 64% of all human vector-borne infection notifications since 1991 [14]. There are averages of approximately 5,000 and 1,500 notified cases of RRV and BFV, respectively, in Australia annually [14]. RRV and BFV are clinically similar, ranging from asymptomatic, to relatively mild symptomatic presentations such as fever and rash, and in more severe disease, polyarthralgia or arthritis [12,15].

Reports of RRV in the PICTs date back to the 1960’s, however until recently, RRV was thought to be endemic only in Australia [16]. RRV seroprevalence has been reported recently outside Australia, including French Polynesia and American Samoa [17,18]. While only a single BFV isolate has been obtained outside Australia, from Papua New Guinea (PNG), BFV is believed to have circulated in PNG long-term [19].

**Fig 1:**
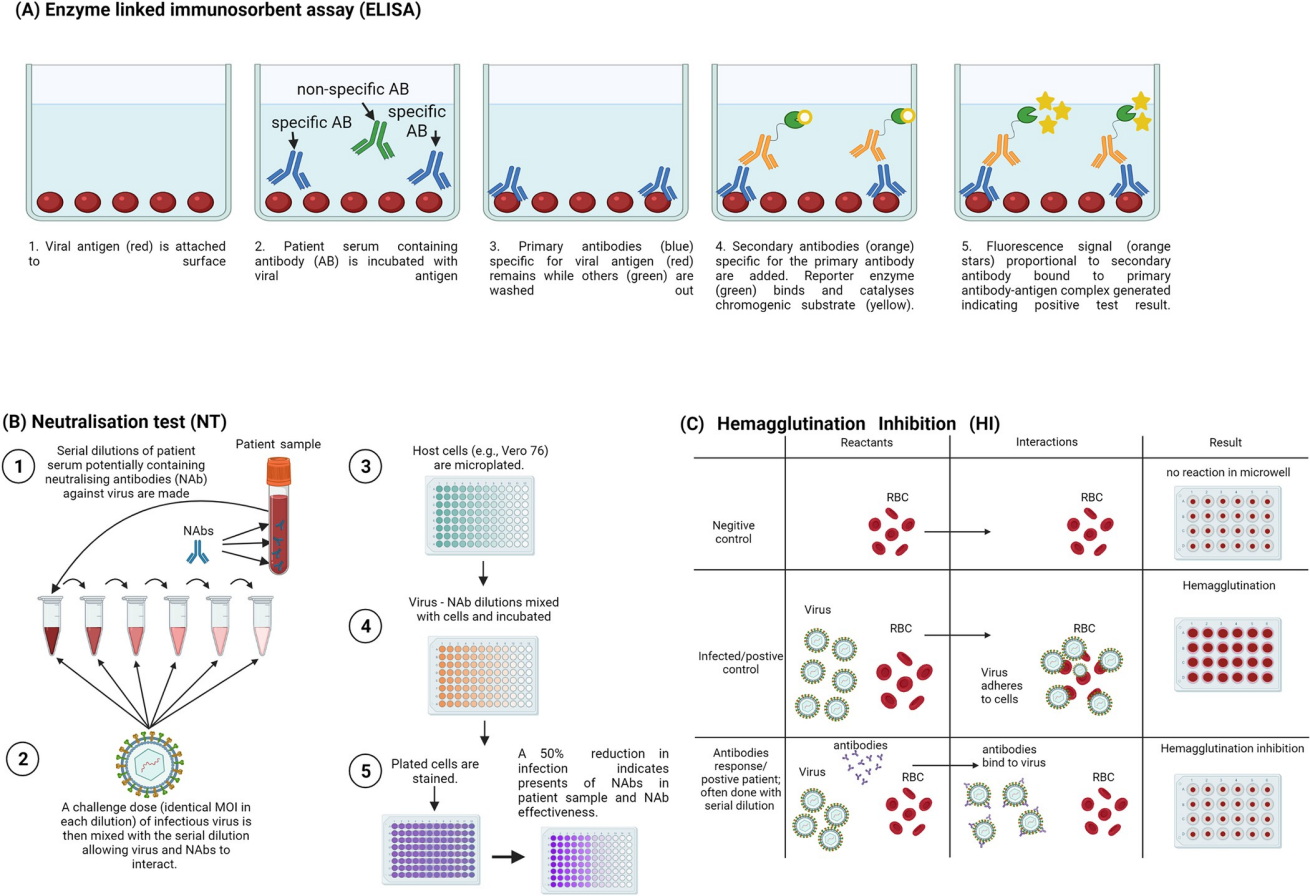
Schematic representation of commonly used serological assays. The figure shows ELISA (A), NT (B) and HI (C) serological assays commonly used to identify anti-arboviral antibodies. Created using BioRender.

Serological assays are used to assess an individual’s exposure and immunity to a given pathogen by measuring antibodies in the blood. The most common serological assays for DENV, RRV and BFV are immunoassays such as the enzyme-linked immunosorbent assay (ELISA), immunofluorescent assays (IFA) or next generation microsphere immunoassay (MIA). These tests have the added benefit of being able to distinguish between immunoglobulin M (IgM) and immunoglobulin G (IgG) antibodies in serum samples. Other commonly used serological assays include the neutralisation test (NT) and hemagglutination inhibition test (HI; Fig 1).

The ELISA is the most used immunoassay and is a highly specific method to determine the presence of antibodies in serum. In ELISAs an antigen (e.g. viral protein) is immobilized on a solid surface followed by the addition of serum containing antibodies to be measured (either IgM or IgG). The antigen-specific antibody, if present in the serum, then binds forming an antigen-antibody complex, which is then detected by a secondary antibody containing an attached reporter (i.e., a fluorophore). In recent years this technology has been further improved through the development of next generation multiplex assays such as the MIA [20].

A neutralisation test (NT) assesses the level of neutralising antibodies in a serum sample by measuring the level of protection from infection. A known titre of pathogen (in this case virus) is mixed with dilutions of serum allowing the antibodies present in the serum to bind and inactivate the pathogen. An infectivity assay is then performed (such as plaque assay or TCID50), and the level of neutralising antibodies calculated by comparing between samples and controls. This is more commonly conducted *in vitro* but can also be performed *in vivo*.

A hemagglutination inhibition (HI) assay relies on the ability of glycoproteins on a pathogen to attach to the surface of red blood cells (RBC) and bind the RBC together (agglutinate). In the presence of specific host antibodies, the pathogen no longer agglutinates the RBC, and this is indicative of a positive serological response. The highest dilution of serum that prevents hemagglutination is called the HI titre.

A positive immunoassay IgM test is consistent with recent virus infection, whereas positive immunoassay IgG usually indicates past infection. This is because IgG antibodies are generated later than IgM antibodies [21]. The NT and HI are commonly used as confirmatory tests for DENV, RRV and BFV.

Serosurveys cannot be pooled across methods or populations, but it is critical to review assessments of past and current circulation of DENV, RRV and BFV. Consequently, we systematically reviewed serosurveys for these three viruses in Australia and the PICTs. We aim to identify trends in seroprevalence and critique study designs by synthesising these studies.

## Methods

### Ethics statement

This systematic review followed the Preferred Reporting Items for Systematic Reviews and Meta- Analyses (PRISMA) guidelines (S1 PRISMA Checklist, S1 Data) [22].

### Search strategy and selection criteria

We searched Web of Knowledge Thomson Reuters, PubMed, Google Scholar and Science Direct for original research articles on the seroprevalence of DENV, RRV and BFV among humans in Australia and PICTs from 1900 to February of 2021. The PICTs includes the Commonwealth of the Northern Mariana Islands, the Federated States of Micronesia, Fiji, French Polynesia, Kiribati, the Marshall Islands, Nauru, New Caledonia, New Zealand, Palau, Papua New Guinea, Solomon Islands, Tonga, Tuvalu, Vanuatu, and Wallis and Futuna Islands. The following combinations of keywords were used to search the literature: ‘Ross River virus’, ‘Ross River fever’, ‘Barmah Forest virus’, ‘Dengue virus’, ‘sero-prevalence’, ‘seroprev*’, ‘serosurv*’, ‘serolog*’, ‘sero-epidemiology’, ‘seroepidemiology’, ‘serum’, ‘antigen’, ‘antibod*’, ‘hemagglutination’, ‘hemagglutination inhibition’, ‘IgM’, ‘IgG’, ‘immunoglobulin’, ‘neutralization’, ‘neutralisation’, ‘assay’, ‘enzyme-linked immunosorbent assay’, ‘ELISA’, ‘patholog*’, ‘and’, ‘or’, ‘Australia’ and names of specific countries in the PICT listed above. We used the asterisk * operator as a wildcard to search for all possible variations of a keyword. We included serological studies of RRV, DENV, or BFV using immunoassays (ELISA, MIA, and IFA), NT or HI tests. The geographical area studied was limited to Australia and the PICTs. Case reports, comments and book chapters were excluded as these are not reliable sources for measuring and identifying spatiotemporal trends in population level human seroprevalences. The modelling studies, genetic analyses, retrospective analyses of outbreaks, studies focusing only on optimising methods, studies conducted on non-human species and non-English records were excluded. We also searched reference lists of selected papers to identify additional articles. All the retrieved articles were screened based on inclusion and exclusion criteria (S1 Fig).

### Data extraction, synthesis and visualisation

For each record we extracted the author, study year(s), research area(s), risk factors (age or/and sex), virus(es) investigated, serological method(s) used, method of sample collection, number of samples tested, and the number of serologically positive samples. Data were extracted by each record for a single virus. All the data were extracted by three authors (ETM, WQ, and EXYL). If there were disagreements, a fourth reviewer (DH) decided, which was then agreed with by the other authors. Data synthesis and visualisation was done using statistical methods in GraphPad Prism v9.2.0. To determine how sampling strategy influenced seroprevalence, a Student’s t-test (Mann Whitney Non Parametric) and the One-way ANOVA performed using the Bonferroni’s Multiple Comparisons test were implemented in GraphPad Prism. A chi-squared test was also conducted to determine the association between the serology method and time.

## Results

Among 1,920 studies identified, 41 met our inclusion criteria (S1 Fig) and these reported 78 serosurveys (Table 1) [16, 23–52]. Serosurveys spanned 66 years (1951–2017) and included 62,327 human samples. The majority were tested for RRV (n = 34,444), followed by BFV (n = 14,445) and DENV (n = 13,438).

**Table 1:** Included studies and seroprevalences reported.

Study	Study period	Study design	Research Area*	Country	Method	Type of test^	Confirmatory test	Total tests	Positive tests	Seroprevalence (%)
	***Dengue virus***							***13*,*438***	***3*,*931***	***61*.*8% (median)***
McBride, 1998 (1) [40]	1993	CT	Charters Towers (QLD)	Australia	ELISA(IgG)	PanBio	-	997	614	61.8
McBride, 1998 (2) [40]	1993	CT	Charters Towers (QLD)	Australia	NT and (HI or ELISA)	In-house	-	624	399	63.9
Darcy, 2001 [41]	1994–1995	RS	Honiara	Solomon Islands	ELISA(IgG)	PanBio	ELISA	515	202	39.2
Ratnam, 2012 [44]	2007–2010	RS	Victoria	Australia	ELISA(IgG)	PanBio	ELISA	387	20	5.17
Faddy, 2013 (1) [45]	2008–2009	RS	Queensland	Australia	ELISA(IgM)	PanBio, Standard Diagnostics	ELISA	5453	12	0.2
Faddy, 2013 (2) [45]	2008–2009	RS	Queensland	Australia	ELISA(IgG)	PanBio	-	1548	146	9.4
Faddy, 2013 (3) [45]	2008–2009	RS	Melbourne	Australia	ELISA(IgG)	PanBio	-	457	31	6.8
Duncombe, 2013 [53]	2010	CT	American Samoa	American Samoa	ELISA(IgG)	PanBio	-	794	759	95.6
Aubry, 2015 [17]	2011–2013	RS	French Polynesia	French Polynesia	ELISA(IgG)	In-house	-	593	476	80.3
Luang-Suarkia, 2017 [54]	1959–1963	RS	Papua New Guinea	Papua New Guinea	NT	-	-	123	59	48.0
Aubry, 2018 [49]	2014	RS	French Polynesia	French Polynesia	ELISA(IgG)	In-house	-	672	473	70.4
Aubry, 2018 [49]	2015	RS	French Polynesia	French Polynesia	MIA(IgG)	In-house	-	700	582	83.1
Darcy, 2020 [51]	2016	CT	Honiara and Gizo	Solomon Islands	ELISA(IgG)	Vircell	-	188	158	84.0
	***Ross River virus***							***34*,*444***	***6*,*549***	***18*.*95% (median)***
Doherty, 1966 (1) [24]	1957–1964	RS	Queensland	Australia	HI	-	NT	636	316	49.7
Doherty, 1966 (2) [24]	1957–1964	RS	Queensland	Australia	NT	-	-	308	139	45.1
Doherty, 1968 [25]	1951–1955	RS	Near Innisfail	Australia	HI	-	-	284	128	45.1
Clarke, 1973 [26]	1965–1971	RS	New South Wales	Australia	HI	-	-	1278	215	16.8
Doherty, 1973 (1) [27]	1967	RS	Queensland	Australia	HI	-	-	507	239**	47.1
Doherty, 1973 (2) [27]	1966–1971	RS	Eastern Queensland	Australia	HI	-	-	1463	614	42.0
Doherty, 1973 (3) [27]	1969	RS	Northern Territory	Australia	HI	-	-	198	98	49.5
Seglenieks, 1974 [55]	1971 (during epidemic)	CT	South Australia	Australia	HI	-	-	109	109	100.0
Seglenieks, 1974 [55]	1971 (post epidemic)	RS	South Australia	Australia	HI	-	-	104	21	20.2
Tesh,1975 (1) [28]	1960, 1965–1966	RS	Solomon Islands	Solomon Islands	NT	-	-	604	40	6.6
Tesh, 1975 (2) [28]	1963	RS	New Caledonia	New Caledonia	NT	-	-	72	0	0.0
Tesh, 1975 [28]	1960, 1965, 1968–1969	RS	Papua New Guinea	Papua New Guinea	NT	-	-	604	204	33.8
Tesh, 1975 [28]	1962	RS	Tula	American Samoa	NT	-	-	30	0	0.0
Liehne, 1976 [29]	NA	RS	Kimberley area	Australia	HI	-	-	441	181	41.0
Stallman, 1976 (1) [30]	1974–1975	CT	Queensland	Australia	HI	-	-	2677	206	7.7
Stallman, 1976 (2) [30]	1974–1975	CT	Northern Territory	Australia	HI	-	-	53	8	15.1
Mudge, 1977 [56]	1976	CT	Riverland area, South Australia	Australia	HI	-	-	57	45	78.9
Kanamitsu, 1979 [57]	1972-possibly 1978	RS	Queensland	Australia	HI	-	-	132	41	31.1
Aaskov, 1981 [16]	1978–1979	RS	Queensland	Australia	HI	-	IFA	345	77	22.3
Aaskov, 1981 [23]	1979	CT	Western Viti Lev	Fiji	HI	-	-	418	386	92.3
Cloonan, 1982 [31]	1979–1980	RS	New South Wales	Australia	ELISA(IgG)	In-house	-	468	31	6.6
Fraser, 1986 [32]	1974–1975	RS	Echuca	Australia	HI	-	-	739	90	12.2
Campbell, 1989 [35]	1988	RS	Victoria	Australia	ELISA(IgG)	ND	NT	523	130	24.9
Campbell, 1989 [35]	1988–1989	CT	Victoria	Australia	ELISA(IgG)	ND	NT	207	20	9.7
Phillips, 1990 (1) [36]	1988–1989	CT	Queensland	Australia	HI	-	NT	2010	636	31.6
Humphrey-Smith, 1991 (1) [37]	NA	RS	Heron Island	Australia	NT	-	-	101	9	8.9
Hawkes, 1993 [38]	1981–1982	ET	New South Wales	Australia	HI	-	-	2109	578**	27.4
Weinstein, 1994 [39]	1992	RS	South Australia	Australia	ELISA(IgG)	In-house	HI	4776	395	8.3
Hii, 1997 [58]	1991	RS	Nogolitogo	Papua New Guinea	ELISA(IgG)	ND	-	58	34	59.0
Dodsley, 2001 [42]	1998	RS	Western Australia	Australia	HI	-	NT	806	58**	7.2
Allchin, 2003 [59]	2001	RS	Western Sydney, New South Wales	Australia	ELISA (IgM)	ND	ELISA	325	5	1.5
Allchin, 2003 [59]	2001	RS	Western Sydney, New South Wales	Australia	ELISA (IgG)	ND	Unclear	325	11	3.4
Faddy, 2015 (1) [46]	2011	ET	Queensland	Australia	ELISA(IgM)	PanBio	IFA	4168	37	0.9
Faddy, 2015 (2) [46]	2011	ET	Murray Valley	Australia	ELISA(IgM)	PanBio	IFA	946	9	1.0
Faddy, 2015 (3) [46]	2011	ET	Hobart	Australia	ELISA(IgG)	PanBio	HI	355	19	5.4
Faddy, 2015 (4) [46]	2011	ET	Adelaide	Australia	ELISA(IgG)	PanBio	HI	359	13	3.6
Faddy, 2015 (5) [46]	2011	ET	Queensland	Australia	ELISA(IgG)	PanBio	HI	694	123	17.7
Faddy, 2015 (6) [46]	2011	ET	Darwin	Australia	ELISA(IgG)	PanBio	HI	354	54	15.3
Faddy, 2015 (7) [46]	2011	ET	Melbourne	Australia	ELISA(IgG)	PanBio	HI	359	3	0.8
Faddy, 2015 (8) [46]	2011	ET	Perth	Australia	ELISA(IgG)	PanBio	HI	358	10	2.8
Faddy, 2015 (9) [46]	2011	ET	Sydney	Australia	ELISA(IgG)	PanBio	HI	360	16	4.4
Aubry, 2015 [47]	2011–2013	RS	French Polynesia	French Polynesia	ELISA (IgG)	In-house	ELISA	593	204	34.4
Lau, 2017 [18]	1979–1980	RS	American Samoa	American Samoa	ELISA(IgG)	In-house		196	145	74.0
Aubry, 2017 [48]	2014–2015	RS	French Polynesia	French Polynesia	ELISA(IgG) and MIA	In-house		1372	197	14.4
Gyawali, 2019 [52]	2015	CT	Queensland	Australia	NT	-		100	41	41.0
Gyawali, 2019 [52]	2015	CT	Queensland	Australia	IFA (IgM)	-		32	3	9.4
Aubry, 2019 (1) [50]	2013	RS	Fiji	Fiji	MIA (IgG)	-		778	362	46.5
Aubry, 2019 and 2020 [50]	2015	RS	Central Division	Fiji	MIA (IgG)	-		333	124	37.2
Aubry, 2020 [60]	2017	RS	Central Division	Fiji	MIA (IgG)	-		320	125	39.1
	***Barmah Forest virus***							***14*,*445***	***348***	***1*.*95% (median)***
Vale, 1986 [33]	1980–1985	RS	New South Wales	Australia	NT	-	-	468	22	4.7
Hawkes, 1987 [34]	NA	RS	New South Wales	Australia	ELISA(IgG) and HI	In-house	HI	3540	70	2.0
Phillips, 1990 (2) [36]	1988–1989	CT	Queensland	Australia	HI	-	-	2010	130	6.5
Humphrey-Smith, 1991 (2) [37]	NA	RS	Heron Island	Australia	NT	-	Duplicate NTs run in parallel	101	2	2.0
Johansen, 2005 [43]	1991–1992, 1995	CT	South-western Western Australia	Australia	NT	-	-	322	3	0.9
Faddy, 2015 (10) [46]	2011	ET	Queensland	Australia	ELISA(IgM)	PanBio	IFA	4087	43	1.1
Faddy, 2015 (11) [46]	2011	ET	Murray Valley	Australia	ELISA(IgM)	PanBio	IFA	946	18	1.9
Faddy, 2015 (12) [46]	2011	ET	Hobart	Australia	ELISA(IgG)	PanBio	HI	355	3	0.9
Faddy, 2015 (13) [46]	2011	ET	Adelaide	Australia	ELISA(IgG)	PanBio	HI	359	5	1.4
Faddy, 2015 (14) [46]	2011	ET	Queensland	Australia	ELISA(IgG)	PanBio	HI	694	9	1.3
Faddy, 2015 (15) [46]	2011	ET	Darwin	Australia	ELISA(IgG)	PanBio	HI	354	13	3.7
Faddy,2015 (16) [46]	2011	ET	Melbourne	Australia	ELISA(IgG)	PanBio	HI	359	1	0.3
Faddy, 2015 (17) [46]	2011	ET	Perth	Australia	ELISA(IgG)	PanBio	HI	358	5	1.4
Faddy, 2015 (18) [46]	2011	ET	Sydney	Australia	ELISA(IgG)	PanBio	HI	360	11	3.1
Gyawali, 2019 [52]	2015	CT	Queensland	Australia	NT	-	-	100	9	9.0
Gyawali, 2019 [52]	2015	CT	Queensland	Australia	IFA (IgM)	-	-	32	4	12.5

Abbreviations: DENV = Dengue virus, RRV = Ross River virus, BFV = Barmah Forest virus,— = Not Applicable, ND = Not disclosed, HI = Hemagglutination Inhibition test, NT = Neutralisation test, ELISA = Enzyme-Linked Immunosorbent Assay, CT = clinically targeted, ET = environmentally targeted, RS = random sampling.

*If the research area includes more than one area, a larger area containing these areas was used.

**The number of positive tests were estimated from rates reported in figures from these papers.

^Type of test refers to commercial source or in house for the ELISA-based tests, dash (-) indicates not applicable. Seroprevalence reported in “Aubry, 2019” in 2015 and “Aubry, 2020” in 2015 is identical therefore this was counted as one serosurvey.

Only seven studies mentioned the survey designs (of which four were in a response to part of an outbreak investigation and utilised different methods) including a repeated cross-sectional survey, a cohort study and a random serosurvey. Twenty-one studies reported sampling methods, among these, nine were blood donor surveys. Seven studies selected samples from residents or high-risk populations, and the remaining six collected samples from travellers or patients and a combination of sources. Surveys were conducted in Australia, Papua New Guinea, American Samoa, Solomon Islands, New Caledonia, Fiji, and French Polynesia (Table 2).

**Table 2:** Summary of sampling methods, study designs and locations.

Virus	Sampling method	Number of serosurveys	Study Design				Location
			Case control	Cohort	Cross sectional	Unknown	
DENV	CT	4	-	-	4	-	Australia and PICT
	ET	-	-	-	-	-	-
	RS	9	-	7	2	-	Australia and PICT
RRV	CT	9	-	4	5	-	Australia and PICT
	ET	10	10	-	-	-	Australia
	RS	30	-	16	12	2	Australia and PICT
BFV	CT	4	-	2	2	-	Australia
	ET	9	9	-	-	-	Australia
	RS	3	-	1	-	2	Australia

NB: "Clinically targeted (CT)" = samples collected that target clinical presentations of a defined outbreak location and time; “Environmentally Targeted (ET)” = samples that were collected covering a spatiotemporal region with a specific climactic pattern such as rainfall, temperature, season and, “Random sampling (RS)” = no specific clinical or environmental bias in sampling meaning, generally these are studies that have assessed the seroprevalence in the general population of a region.

### Reported seroprevalence for DENV, RRV and BFV

There was a wide range in Ross River virus (0.0% to 100% Table 1) and DENV (0.2% to 95.6%, Table 1) seropositivity. The seroprevalence range for BFV was smaller (0.3% to 12.5%, Table 1). Dengue virus had the highest median seroprevalence of 61.8%, compared to 19.0% and 2.0% in RRV and BFV, respectively.

There was high variability of reported seroprevalence using the HI, NT, ELISA IgM and ELISA IgG methods for DENV and RRV but low variability for BFV serosurveys across all serological methods (Table 1). The geographical range of surveys for RRV and DENV was also wider covering several countries, whereas BFV was limited to Australia. Studies that measured IgM via ELISA displayed the greatest consistency of prevalence between studies (median values = 0.2%, 1.0%, and 1.5% for DENV, RRV and BFV, respectively).

### Clinical and Environmental association

**Fig 2:**
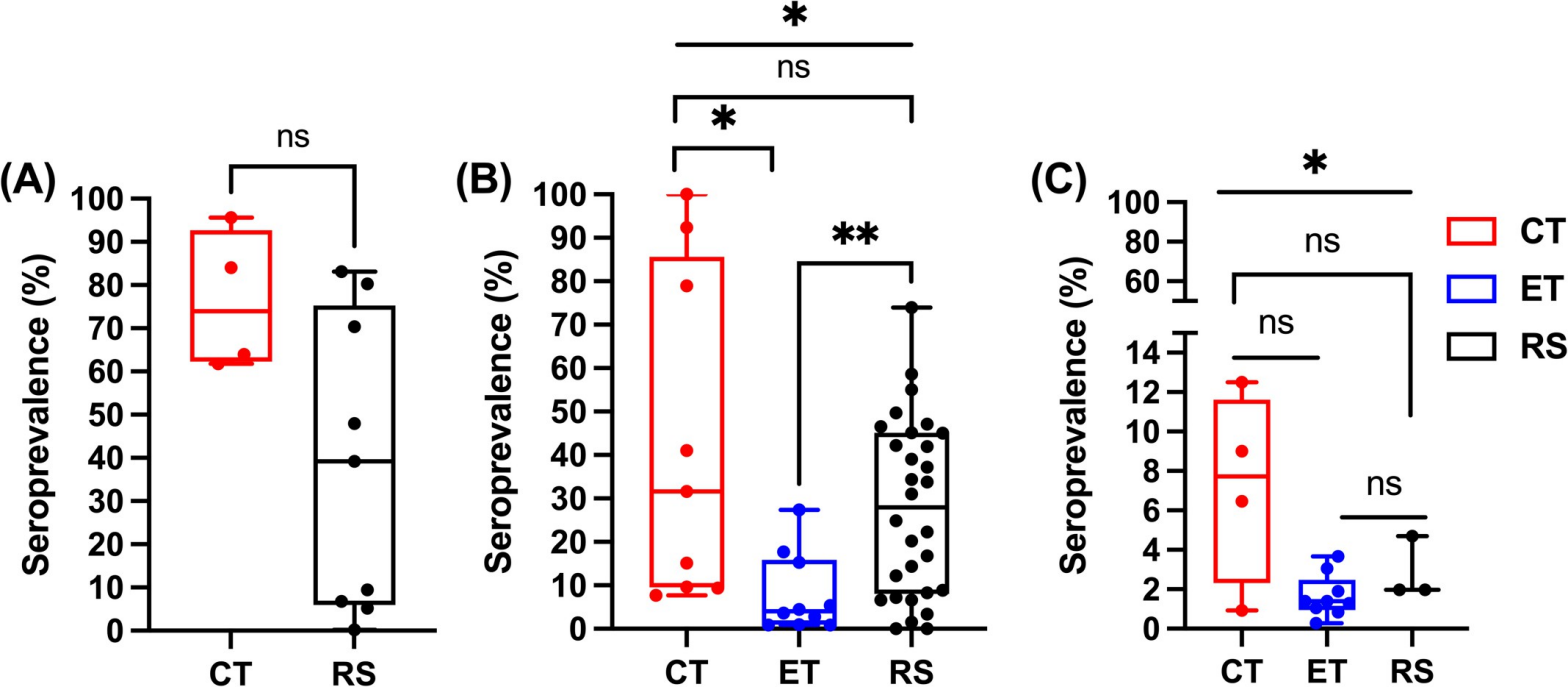
Variance in virus median and seroprevalence ranges by sampling method. The figure shows the high variance in median (%) and seroprevalence ranges from surveys for DENV (A), RRV (B) and BFV (C) based on different sample collection methods: CT = clinically targeted, ET = Environmentally targeted, and RS = Random Sampling. Y-axis = (%) seroprevalence and X-axis = sampling methods (CT, ET, and RS). (A)—analysis performed using t-test (Mann Whitney Non-Parametric); (B) and (C)—analysis done using One-Way ANOVA (Bonferroni’s Multiple Comparisons Test). *p<0.05, **p<0.01, ns = not significant.

Seroprevalence rates varied with sampling method (Fig 2). Here we classify studies broadly into three sample collection strategies. 1) clinically targeted (CT); samples collected that target clinical presentations of a defined outbreak location and time, 2) environmentally targeted (ET); samples that were collected covering a spatio-temporal region with a specific climactic pattern such as rainfall, temperature, season, 3) random sampling (RS); no clinical or environmental purpose in sampling, these are studies that have assessed the seroprevalence in the general population.

A one-way ANOVA performed using the Bonferroni’s Multiple Comparisons test in GraphPad Prism v9.0.2 revealed that sampling strategy influenced seroprevalence for RRV and BFV (*p* < 0.05; Fig 2). Unfortunately, no studies were identified that used an environmentally targeted sample collection strategy for DENV within our inclusion criteria and therefore a similar analysis could not be performed for DENV.

Clinically targeted serosurveys demonstrated the highest seroprevalence across all three viruses. CT DENV serosurveys had the highest median seroprevalence (74%, range: 61.8% - 95.6%), followed by RRV (31.6%, range: 7.7% - 100.0%) and BFV (7.7%, range: 0.9% - 12.5%). Based on the CT sampling method, BFV had the narrowest while RRV had the widest seroprevalence range.

An environmentally targeted approach was only employed for RRV and BFV in Australia. A total of 19 RRV and BFV serosurveys assessed the association of seroprevalence with environmental conditions with RRV having a higher median (4.0%) and wider seroprevalence range (0.83% - 17.7%) than BFV (median = 1.39%, range: 0.3% - 3.7%) and neither demonstrating higher median values than the random sampling approach.

Greater than 50% (42/78) of the serosurveys collected samples randomly (RS). DENV had the highest median seroprevalence (39.2%), followed by RRV (27.9%) and BFV (1.9%) (Fig 2). Similarly, DENV also had the widest seroprevalence range (0.2% - 83.1%), followed by RRV (0.0% - 74.0%) and BFV (1.9% - 4.7%). Further analysis of the studies which utilized a RS approach found location of the population heavily impacted the results. For DENV and RRV, a RS approach was used both in Australia and the PICTs while BFV studies utilized RS only in Australia. All upper extreme outlying (%) seroprevalence values for DENV and RRV were from studies that used the RS approach to collect test samples from PICTs where previous large scale outbreaks have been reported; French Polynesia (DENV; 83.1% and 80.3%) and American Samoa (RRV; 74%) (Fig 2). As shown in Table 2 below, serosurveys could be grouped into different categories based on study designs. Based on this grouping, 38.5% of surveys were cohort studies, 32% were cross-sectional studies, 24.4% were designed as case controls and the design of 5.1% surveys could not be established (Table 2).

### Spatial distribution of serosurveys

Dengue virus serosurveys were performed in four PICT countries (American Samoa, Papua New Guinea, Solomon Islands and French Polynesia), and two Australian states (Queensland and Victoria). The majority of human samples tested for DENV originated from Queensland (52.1%, 7,001/13,438).

The highest seroprevalence was reported during outbreaks of DENV in American Samoa in 2010 (95.6%) and the Solomon Islands in 2016 (84.0%). Random sampling of populations in French Polynesia in 2015 (83.1%), and Charters Towers, Queensland in 1993 (63.9%) where DENV was actively circulating also demonstrated high seroprevalence. Locations with low reported seroprevalence include Queensland between 2008–2009 (0.2%), Victoria between 2007–2010 (5.17%; which targeted people with intention to travel to SE Asia including with prior travel history) and Melbourne between 2008–2009 (6.8%; from blood donors 94% of which reported previous travel to DENV endemic areas).

Ross River virus serosurveys were undertaken in six PICT countries (American Samoa, Papua New Guinea, French Polynesia, Fiji, New Caledonia, and Solomon Islands), and within all Australia states and territories except the Australian Capital Territory. More than thirty-eight percent (38.8%, 13,356/34,444) of the samples were collected in Queensland. The highest seroprevalence was reported on the mainland in South Australia in 1971 (100.0%) followed by Fiji during 1979 (92.3%). The foregoing studies used clinically targeted samples in a known outbreak. Four serosurveys reported seroprevalence values lower than 1% and these were conducted by random sampling in Melbourne (Victoria) in 2011 (0.8%; ELISA-IgG), Queensland in 2011 (0.9%; ELISA-IgM), Tula (American Samoa) in 1962 (0%; NT), and New Caledonia in 1963 (0.0%; NT).

Serological surveys for BFV were all conducted in Australia. Nearly half the samples were collected in Queensland (47.9%, 6,923/14,445), which also reported the highest seroprevalence for BFV in 2015 (12.5%). BFV seroprevalence was also high in New South Wales between 1980 and 1985 (4.7%) in randomly sampled blood donors. Melbourne and Hobart had the lowest seroprevalence with only 0.3% and 0.9%, respectively, from blood donors in 2011.

### Serosurvey methods adopted

**Fig 3:**
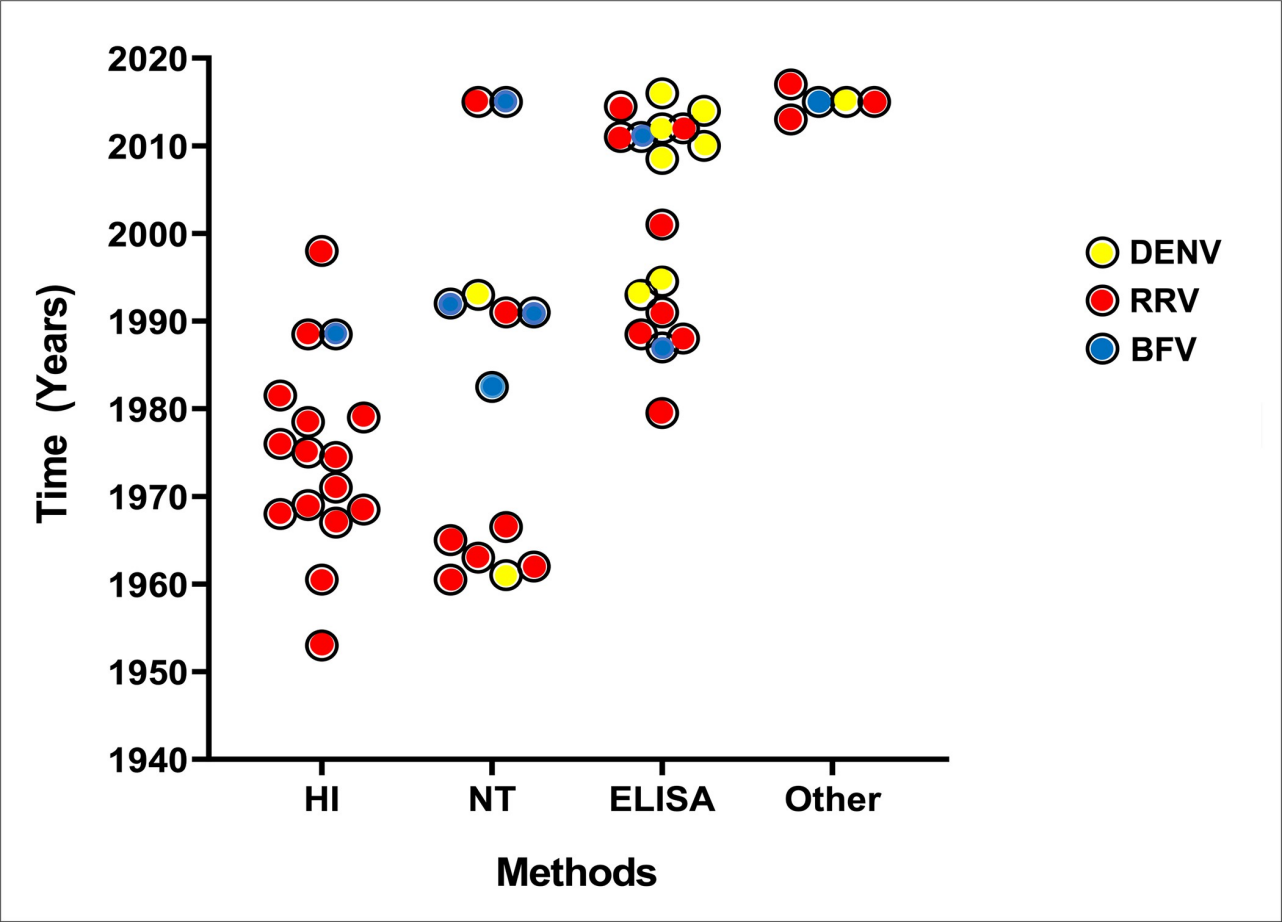
A dot plot showing the relationship between time in years and human seroprevalence for DENV, RRV and BFV reported using the HI, NT, ELISA, and Other (i.e., MIA or IFA) immunoassays. Yellow, red, and blue filled circles = studies reporting human seroprevalences for DENV, RRV and BFV respectively, using the HI, NT, ELISA, and other immune assays. As shown in the figure, more HI and NT were applied in testing before the year 2000 and more ELISA tests were applied after 2000, with a statistically significant association between the serology method and time determined for DENV and RRV. The date of publication was used for Hawkes (1987) [34]–BFV (ELISA); Humphrey-Smith (1991) [37]–BFV (NT) and RRV (NT), and Liehne (1976) [29]–RRV (HI).

Among the 78 serosurveys, two used a combination of serological methods and two used a microsphere-based immunoassay for RRV IgG detection (Table 1). Across the three viruses, serological testing through ELISA (IgG) was most common (32/78 serosurveys; 41.0%), followed by the HI assay (20/78; 25.6%). The majority of DENV (n = 9/13) and BFV (n = 7/16) surveys utilised the ELISA method for IgG antibodies (Table 3). For RRV, the HI assay was the most used (19/49 serosurveys) and ELISA (IgG) was applied in 16 serosurveys (Table 3). No HI and IFA assays were employed for DENV. ELISA (IgM) was used in fewer studies (6/78 = 7.7%) but was the only method used to test more than 5,000 samples for each of the three viruses between 2001 and 2011. There was a trend towards ELISA-based technology over time. More HI and NT tests were applied before 2000 and more ELISA tests were applied after 2000 (Fig 3), with a statistically significant association between the serology method and time determined for DENV (χ^2^ = 33.82, *df* = 1, *p* = 0.0001) and RRV (χ^2^ = 9.35, *df* = 1, *p* = 0.0022).

**Table 3:** Serological methods used to test samples for antibodies against virus.

Serological methods *		DENV	RRV	BFV	Total
ELISA(IgG)	Serosurveys	9	16	7	32
	Samples tested	6,538	11,357	2,839	20,734
	Positive samples	2,879	1,405	47	4,331
ELISA(IgM)	Serosurveys	1	3	2	6
	Samples tested	5,453	5,439	5,033	15,925
	Positive samples	12	51	61	124
IFA (IgM)	Serosurveys	-	1	1	2
	Samples tested	-	32	32	64
	Positive samples	-	3	4	7
MIA (IgG)	Serosurveys	1	3	-	4
	Samples tested	700	1,431	-	2,131
	Positive samples	582	611	-	1,193
HI	Serosurveys	-	19	1	20
	Samples tested	-	14,366	2010	16,376
	Positive samples	-	4,046	130	4,176
NT	Serosurveys	1	7	4	12
	Samples tested	123	1819	991	2,933
	Positive samples	59	433	36	528
NT and (HI or ELISA)	Serosurveys	1	-	-	1
	Samples tested	624	-	-	624
	Positive samples	399	-	-	399
ELISA(IgG) and HI	Serosurveys	-	-	1	1
	Samples tested	-	-	3,540	3,540
	Positive samples	-	-	70	70
Total	Serosurveys	13	49	16	78
	Samples tested	13,438	34,444	14,445	62,327
	Positive samples	3,931	6,549	348	10,828

Abbreviations: DENV = Dengue virus, RRV = Ross River virus, BFV = Barmah Forest virus, ELISA = Enzyme-Linked Immunosorbent Assay, HI = Hemagglutination Inhibition test, IFA = immunofluorescence assay, MIA = microsphere immunoassay, NT = Neutralisation test.

### Reported seroprevalence across age or gender groups

Seventeen RRV studies, three BFV studies and four DENV studies reported seroprevalence across age groups (S1 Table). The majority of the serosurveys analysed reported a positive association of seroprevalence and age group (except studies by Liehne et al., 1976 [29]; Weinstein *et al*., 1994 [39]; Hii *et al*., 1997 [58]; Dodsley et al., 2001 [42] and Faddy et al., 2015 [46]). Eight RRV studies, three BFV studies and three DENV studies reported seroprevalence among males and females and these comprise 14 serosurveys (S2 Table). We observed slightly greater seroprevalences in males compared to females in most serosurveys (10/14), without statistical significance.

## Discussion

Our review presents serological surveys for three important human arbovirus infections across 66 years (1951–2017) for Australia and the Pacific. Information about seroprevalence in the general population is useful for assessing disease risk and transmission patterns. The collation of these data adds an important data resource for arbovirus researchers.

### Seroprevalence of DENV, RRV and BFV

Of the three arboviruses, RRV seropositivity was reported in the greatest number of countries (Australia and three PICTs, Fiji, French Polynesia, and Solomon Islands). RRV has long been considered endemic to Australia, and reservoir hosts were historically thought to be marsupials (such as kangaroos and possums). This dogma has been shifting in more recent years supported by ecological and epidemiological studies on non-human reservoirs. From studies collated here there is evidence RRV has been circulating outside Australia as early as the 1960’s–before a RRV outbreak in the PICTs in 1979. Specifically, Tesh (1975) [28] tested for RRV in the Asia-Pacific and here we report on seropositivity in Papua New Guinea and the Solomon Islands. Zero prevalence from NT assays was reported in surveys conducted in American Samoa and New Caledonia in 1962 and 1963 but from small samples. Though there has been speculation that RRV may be silently circulating in the PICTs, this review highlights that the circulation of RRV in the PICTs is not a recent phenomenon but possibly has been overlooked until now.

Based on ELISA IgG serosurveys, DENV prevalence was greatest in the PICTs. This is to be expected in the context of DENV epidemiology. The primary vectors of DENV are tropical mosquito species *Ae*. *aegypti* and *Ae*. *albopictus*. Without this context, geographical aggregation of DENV serosurvey results may be misleading. For example, DENV seroprevalences reported in Victoria [44,61] were not due to locally acquired infection, but rather returning travellers who had acquired infection elsewhere (most commonly Southeast Asia and the Pacific), where DENV is endemic. Infected travellers potentially pose a threat to local populations. This is particularly important in Northern Queensland where DENV incidence and *Ae*. *aegypti* abundance used to be high before deployment of *Wolbachia* infected mosquitoes [62].

By comparison to RRV and DENV, studies for BFV were few. This is likely due to the lower incidence of BFV in Australia (with reported transmission in the PICTs) and on account of its relatively recent identification. Barmah Forest virus was first isolated in 1974 [63] and there were no serosurveys until 1986 [33]. Cases may have been misdiagnosed as RRV as these viruses share the same geographical distribution, clinical presentations, vectors, and possibly reservoir hosts [12], however to date there is minimal evidence for cross-reactivity between BFV and RRV. The highest seroprevalence of BFV in this study was 12.5%. Infection with an alphavirus within the same serocomplex may protect against the other [64], however as RRV and BFV are classified in distinct serocomplexes, it is unclear if high RRV population immunity limits the number of BFV infections. Why BFV remains relatively rare compared to RRV is currently uncertain. Barmah Forest virus serosurveys have been conducted in all states and territories in Australia but not in the PICTs. We recommend that future serological surveys conducted in the PICTs should also include BFV.

Our study also found statistically significant relationship between the sampling strategy and seroprevalence. Whereas studies conducted using the random sampling approach generally tested more samples, the reported seroprevalences were highest for samples collected using a clinically targeted strategy. We found no association between an environmental targeted sampling strategy and seroprevalence, however it must be noted that there were limited studies that utilised environmental stratifications of seroprevalence data. Several studies have shown relationships between arbovirus seroprevalence and environmental factors [65,66] and therefore further studies for RRV, BFV and DENV in the PICT would help to further elucidate any environmental trends.

### Serological assays

Assay method had a significant influence on estimated seroprevalence rates. For instance, studies which utilised ELISA IgM methods often reported zero or low seroprevalence. Those using ELISA IgG reported the highest seroprevalence rates for the three arboviruses. These methods test different phases in the immune response to viral exposure; generally IgM antibodies are produced immediately after exposure to a pathogen and can only rarely be detected several years post-exposure [15]. While IgG levels rise a few weeks post-infection, antibody are usually detected several years following exposure [15].

More than half of the samples in this review were tested for IgM antibodies in only three studies for DENV, RRV and BFV involving six serosurveys [45,46,59]. The Faddy studies (2013 and 2015) using ELISA IgM analysed donated blood samples collected by the Australian Red Cross [45,46]. It is likely that the low levels of seropositivity detected through ELISA (IgM) in these two studies are due to asymptomatic infections because people diagnosed with arboviral infections may not donate blood until four weeks post-recovery [45,46]. The donors did not inform the Australian Red Cross of any diagnosed arbovirus infection before and after blood donation [45,46]. Due to the nature and specificity of the ELISA (IgM) method, it is best employed for investigating outbreaks. The low seroprevalence using ELISA IgM assays is because when there is not an outbreak the probability of recent exposure to DENV, RRV or BFV is low.

The statistically significant association found between the serology method and time suggests that increased application of ELISA-based immunoassays over traditional NT and HI assays as surveillance tools for RRV, DENV, and BFV. This is not surprising as improvements in ELISA-based assays have improved reliability, sensitivity, and specificity over time [67]. Additionally, ELISA-methods have been validated as a reliable means to diagnose RRV, BFV and DENV for health departments globally. Neutralisation and hemagglutination inhibition tests are typically in-house assays. Neutralisation tests indicate that the antibodies present in the patient samples are likely effective in viral clearance. While serosurveys are important for understanding virus transmission and population dynamics, high-throughput screening of serological samples also allow for the identification of neutralising or highly reactive antibodies which could have applications in targeted antibody-based therapeutics and academic research.

This review aims to analyse population exposure to DENV, RRV and BFV in Australia and the PICTs. Therefore, we collected serology-based studies. Other methods of evaluating human sera samples commonly include quantitative polymerase chain reaction (qPCR). This is a molecular-based technique that allows for the detection and quantification of pathogen genomes present in clinical samples. While such methods are potentially informative in the context of determining virus exposure in the population, they were excluded from this analysis as they fall outside the scope of the study.

### Limitations

Findings from serological surveys must be examined in the context of the clinical, ecological and epidemiological aspects of the pathogen. Among the 1920 studies identified, only 41 met our inclusion criteria.

The main limitation of this review is the discontinuity, spatially and temporally, between studies. Only a few of the participants of these studies were from the general population. The extreme heterogeneity of study populations, serosurvey methods and data collected makes it difficult to aggregate results across studies to distinguish spatial and temporal trends across viruses or locations. Despite this, our systematic review contributes new knowledge on DENV, RRV and BFV transmission and provides important information to improve future seroprevalence studies.

Cross-reactivity of antibodies against the three viruses may influence serological test results, and this is a factor that most research studies included in this review fail to address. Misdiagnosis occurs in serological testing. A recent study attempted to determine the aetiologies of undifferentiated febrile illnesses due to arbovirus infections in Australia [52]. While RRV and BFV are routinely screened due to their prevalence, other often neglected arboviruses circulating in Australia include Alfuy (ALFV), Edge Hill (EHV), Kokobera (KOKV), Kunjin (KUNV), Murray Valley encephalitis (MVEV), Sindbis (SINV), and Stratford (STRV) viruses. Among the IgM-reactive samples from patients diagnosed with infection, the authors found that 12/32 samples demonstrated neutralisation of more than one virus tested [52]. Antibody cross-reactivity in serological testing may produce false-positives. Most ELISA tests were performed with commercial assays developed by one company (PanBio), and these tests likely have similar sensitivity and specificity compared to in-house ELISA assays. This should reduce variation in cut-offs for reporting. Some studies have opted to perform additional confirmatory tests on positive samples detected using alternate serological assays, and this is useful for validating data obtained through in-house assays.

## Conclusion

Serosurveys are an important tool to measure human exposure to arboviruses. They are more powerful when standardised and conducted using highly sensitive and specific diagnostic methods. We aimed to review epidemiologically relevant trends, potential biases and risk factors associated with DENV, RRV and BFV seropositivity in Australia and the PICTs. There is variability in reported seroprevalence, study designs, and methods adopted. Most studies did not conduct statistical analyses to determine the significance of risk factors, or the accuracy of the method used to detect seroconversion. To be able to rationally compare studies and therefore detect statistical significance in meta-analysis, future serosurveys conducted in Australia and the PICTs should be standardised. The emergence of medically important arboviruses in new geographical areas (e.g., Japanese encephalitis virus in India, West Nile virus in North America, and zika virus in Brazil [46]) emphasises the importance of building knowledge of the transmission of these viruses. Serosurveys provide important data to this end. Knowledge of DENV, RRV and BFV transmission in Australia and the PICTs will translate to a better understanding of other arboviruses.

## Supporting information

S1 PRISMA Checklist Checklist of items included in our systematic review according to the Preferred Reporting Items for Systematic Reviews and Meta-Analyses (PRISMA) statement recommendations. (DOC)

S1 Fig Flowchart. Flow diagram of the systematic search strategy used to identify and include studies into our systematic review of reported DENV, RRV and BFV seroprevalence among humans in Australia and the PICTs.
(TIF)

S1 Data Metadata and results of data synthesis for all studies included. (XLSX)

S1 Table Studies reported seroprevalences across age groups. (DOCX)

S2 Table Studies reported seroprevalences across gender groups. (DOCX)

## References

[1] Shaw WR and Catteruccia F. Vector biology meets disease control: using basic research to fight vector-borne diseases. *Nat Microbiol*. 2018:1. doi: 10.1038/s41564-018-0214-7 30150735 PMC6437764

[2] Weaver SC. Prediction and prevention of urban arbovirus epidemics: A challenge for the global virology community. *Antiviral Res*. 2018;156:80–4. doi: 10.1016/j.antiviral.2018.06.009 29906475 PMC6082388

[3] Wilder-Smith A, Gubler DJ, Weaver SC, Monath TP, Heymann DL, Scott TW. Epidemic arboviral diseases: priorities for research and public health. *Lancet Infect Dis*. The Lancet Infectious Diseases. 2017;17(3):e101—e6. doi: 10.1016/S1473-3099(16)30518-7 28011234

[4] Calvez E, Mousson L, Vazeille M, O’Connor O, Cao-Lormeau VM, Mathieu-Daude F, et al., Zika virus outbreak in the Pacific: Vector competence of regional vectors. *PLoS Negl Trop Dis*. 2018;12(7):e0006637. doi: 10.1371/journal.pntd.0006637 30016372 PMC6063428

[5] Queensland Health. Dengue: virus, fever and mosquitoes 2020 [cited 2020 20 Feb]. Available from: https://www.health.qld.gov.au/clinical-practice/guidelines-procedures/diseases-infection/diseases/mosquito-borne/dengue/virus-fever.

[6] Halstead SB. Dengue virus–mosquito interactions. *Annu Rev Entomol*. 2008;53:273–91. doi: 10.1146/annurev.ento.53.103106.093326 17803458

[7] Akter R, Naish S, Hu W, Tong S. Socio-demographic, ecological factors and dengue infection trends in Australia. *PLoS One*. 2017;12(10):e0185551. doi: 10.1371/journal.pone.0185551 28968420 PMC5624700

[8] Bandyopadhyay S, Lum LC, Kroeger A. Classifying dengue: a review of the difficulties in using the WHO case classification for dengue haemorrhagic fever. *Trop Med Int Health*. 2006;11(8):1238–55. doi: 10.1111/j.1365-3156.2006.01678.x 16903887

[9] Fitzpatrick C, Haines A, Bangert M, Farlow A, Hemingway J, Velayudhan R. An economic evaluation of vector control in the age of a dengue vaccine. *PLoS Negl Trop Dis*. 2017;11(8):e0005785. doi: 10.1371/journal.pntd.0005785 28806786 PMC5573582

[10] Canyon DV. Historical analysis of the economic cost of dengue in Australia. *J Vector Borne Dis*. 2008;45:245–8. . 18807382

[11] International Federation of Red Cross and Red Crescent Societies. Solomon Islands: Dengue outbreak Emergency Plan of Action (EPoA) DREF Operation MDRSB005. 2016.

[12] Jacups SP, Whelan PI, Currie B. Ross River virus and Barmah Forest virus infections: A review of history, ecology, and predictive models, with implications for tropical northern Australia. *Vector Borne and Zoonotic Dis*. 2008;8(2):283–97. doi: 10.1089/vbz.2007.0152 18279007

[13] Stephenson EB, Peel AJ, Reid SA, Jansen CC, McCallum H. The non-human reservoirs of Ross River virus: a systematic review of the evidence. *Parasit Vectors*. 2018;11(1):188. doi: 10.1186/s13071-018-2733-8 29554936 PMC5859426

[14] Department of Heath, Commonwealth Government of Australia. National Notifiable Disease Surveillance System. 2020. [cited 2020 12 Jun]. Available from: https://www1.health.gov.au/internet/main/publishing.nsf/Content/cda-surveil-nndss-nndssintro.htm

[15] Harley D, Sleigh A, Ritchie S. Ross River virus transmission, infection, and disease: a cross-disciplinary review. *Clin Microbiol Rev*. 2001;14(4):909–32. doi: 10.1128/CMR.14.4.909-932.2001 11585790 PMC89008

[16] Aaskov JG, Ross P, Davies CE, Innis MD, Guard RW, Stallman ND, Tucker M. Epidemic polyathritis in northeastern Australia, 1978–1979. *Med J Aust*. 1981;2(1):17–19. . 7278766

[17] Aubry M, Finke J, Teissier A, Roche C, Broult J, Paulous S et al. Silent Circulation of Ross River Virus in French Polynesia. *Int J Infect Dis*. 2015;37:19–24. doi: 10.1016/j.ijid.2015.06.005 26086687

[18] Lau C, Aubry M, Musso D, Teissier A, Paulous S, Despres P et al. New evidence for endemic circulation of Ross River virus in the Pacific Islands and the potential for emergence. *Int J Infect Dis*. 2017;57:73–6. doi: 10.1016/j.ijid.2017.01.041 28188934

[19] Michie A, Ernst T, Chua I-LJ, Lindsay MDA, Neville PJ, Nicholson J, et al. Phylogenetic and Timescale Analysis of Barmah Forest Virus as Inferred from Genome Sequence Analysis. *Viruses*. 2020;12(7):732. doi: 10.3390/v12070732 32640629 PMC7412159

[20] Basile AJ., Horiuchi K, Panella AJ, Laven J, Kosoy O, Lanciotti RS, et al. Multiplex microsphere immunoassays for the detection of IgM and IgG to arboviral diseases. *PLoS One* 2013;8(9):e75670. doi: 10.1371/journal.pone.0075670 24086608 PMC3783417

[21] World Health Organization. Dengue and severe dengue 2020 [cited 2020 23 Jun]. Available from: https://www.who.int/news-room/fact-sheets/detail/dengue-and-severe-dengue.

[22] Moher D, Liberati A, Tetzlaff J, Altman DG, The PG. Preferred Reporting Items for Systematic Reviews and Meta-Analyses: The PRISMA Statement. *PLOS Med*. 2009;6(7):e1000097. doi: 10.1371/journal.pmed.1000097 19621072 PMC2707599

[23] Aaskov J, Mataika J, Lawrence G, Rabukawaqa V, Tucker M, Miles J, et al. An epidemic of Ross River virus infection in Fiji, 1979. *Am J Trop Med Hyg*. 1981;30(5):1053–9. doi: 10.4269/ajtmh.1981.30.1053 7283004

[24] Doherty RL, Gorman B, Whitehead R, Carley J. Studies of Arthropod-borne Virus Infections in Queensland: V. Survey of Antibodies to Group A Arboviruses in Man and other Animals. *Aust J Exp Biol Med Sci*. 1966;44(4):365–78. doi: 10.1038/icb.1966.35 6007741

[25] Doherty R, Standfast H, Wetters E, Whitehead R, Barrow G, Gorman B. Virus isolation and serological studies of arthropodborne virus infections in a high rainfall area of north queensland. *Trans R Soc Trop Med Hyg*. 1968;62(6):862–7. doi: 10.1016/0035-9203(68)90014-x 4389154

[26] Clarke JA, Marshall ID, Gard G. Annually recurrent epidemic polyarthritis and Ross River virus activity in a coastal area of New South Wales: I. Occurrence of the disease. *Am J Trop Med Hyg*. 1973;22(4):543–50. doi: 10.4269/ajtmh.1973.22.543 4197954

[27] Doherty RL. Surveys of haemagglutination-inhibiting antibody to arboviruses in aborigines and other population groups in northern and eastern Australia, 1966–1971. *Trans R Soc Trop Med Hyg*. 1973;67(2):197–205. doi: 10.1016/0035-9203(73)90144-2 4784056

[28] Tesh RB, Gajdusek DC, Garruto RM, Cross JH, Rosen L. The distribution and prevalence of group A arbovirus neutralizing antibodies among human populations in Southeast Asia and the Pacific islands. *Am J Trop Med Hyg*. 1975;24(4):664–75. doi: 10.4269/ajtmh.1975.24.664 1155702

[29] Liehne C, Stanley N, Alpers M, Paul S, Liehne P, Chan K. Ord River arboviruses—serological epidemiology. *Aust J Exp Biol Med Sci*. 1976;54(5):505–12. doi: 10.1038/icb.1976.51 1021093

[30] Stallman ND, Wiemers MA, Bourke ATC. Serology in diagnosis and surveys of man. *Arbovirus Res Aust*. 1976;1:46–64.

[31] Cloonan M, O’Neill B, Vale T, Carter I, Williams J. Ross River virus activity along the south coast of New South Wales. *Aust J Exp Biol Med Sci*. 1982;60(6):701–6. doi: 10.1038/icb.1982.71 6303286

[32] Fraser J, Christie D, Gust I, White J, Leach R, Macaulay E, et al. Arbovirus infection in a murray valley community. *Aust N Z J Med*. 1986;16(1):52–7. doi: 10.1111/j.1445-5994.1986.tb01116.x 3010928

[33] Vale T, Carter I, McPhie K, James GS, Cloonan M. Human arbovirus infections along the south coast of New South Wales. *Aust J Exp Biol Med Sci*. 1986;64(3):307–9. doi: 10.1038/icb.1986.32 3767767

[34] Hawkes RA, Nairn HM, Myrick BM, Ramsay LG. Barmah Forest virus infections in humans in New South Wales. *Med J Aust*. 1987;146(11):569–73. doi: 10.5694/j.1326-5377.1987.tb120416.x 3039324

[35] Campbell J, Aldred J, Davis G, editors. Some aspects of the natural history of Ross River virus in south east Gippsland, Victoria. Arbovirus research in Australia Proceedings Fifth Symposium, August 28-September 1, 1989, Brisbane, Australia; 1989: CSIRO Division of Tropical Animal Production.

[36] Phillips DA, Murray JR, Wiemers MA, Aaskov JG. Clinical and subclinical Barmah Forest virus infection in Queensland. *Med J Aust*. 1990;152(9):463–6. doi: 10.5694/j.1326-5377.1990.tb125304.x 2166224

[37] Humphery-Smith I, Cybinski D, Byrnes K, St George T. Seroepidemiology of arboviruses among seabirds and island residents of the Great Barrier Reef and Coral Sea. *Epidemiol Infect*. 1991;107(2):435–40. doi: 10.1017/s0950268800049086 1657626 PMC2272077

[38] Hawkes RA, Pamplin J, Nairn HM, Boughton CR. Arbovirus infections of humans in high-risk areas of south-eastern Australia: a continuing study. *Med J Aust*. 1993;159(3):159–62. doi: 10.5694/j.1326-5377.1993.tb137778.x 8393128

[39] Weinstein P, Cameron S, Worswick D, McIntyre A. Human sentinels for arbovirus surveillance and regional risk classification in South Australia. *Med J Aust*. 1994;160(8):494–9. . 8170425

[40] McBride W, Mullner H, LaBrooy JT, Wronski I. The 1993 dengue 2 epidemic in North Queensland: a serosurvey and comparison of hemagglutination inhibition with an ELISA. *Am J Trop Med Hyg*. 1998;59(3):457–61. doi: 10.4269/ajtmh.1998.59.457 9749644

[41] Darcy A, Clothier H, Phillips D, Bakote’e B, Stewart T. Solomon Islands dengue seroprevalence study-previous circulation of dengue confirmed. *P N G Med J*. 2001;44(1/2):43–7. . 12418677

[42] Dodsley N, Broom A, Smith D, Plant A, Lindsay M. Ross River virus: determining the prevalence in the south west of Western Australia. *Arbovirus Res Aust*. 2001;8:122–5.

[43] Johansen CA, Mackenzie JS, Smith DW, Lindsay MD. Prevalence of neutralising antibodies to Barmah Forest, Sindbis and Trubanaman viruses in animals and humans in the south-west of Western Australia. *Aust J Zool*. 2005;53(1):51–8. doi: 10.1071/ZO03042

[44] Ratnam I, Black J, Leder K, Biggs BA, Matchett E, Padiglione A, et al. Incidence and seroprevalence of dengue virus infections in Australian travellers to Asia. *Eur J Clin Microbiol Infect Dis*. 2012;31(6):1203–10. Epub 2011/10/11. doi: 10.1007/s10096-011-1429-1 21983919

[45] Faddy HM, Seed CR, Fryk JJ, Hyland CA, Ritchie SA, Taylor CT, et al. Implications of dengue outbreaks for blood supply, Australia. *Emerg Infect Dis*. 2013;19(5):787. doi: 10.3201/eid1905.121664 23648012 PMC3647514

[46] Faddy H, Dunford M, Seed C, Olds A, Harley D, Dean M, et al. Seroprevalence of antibodies to Ross River and Barmah Forest viruses: Possible implications for blood transfusion safety after extreme weather events. Ecohealth. 2015;12(2):347–53. doi: 10.1007/s10393-014-1005-0 25537629

[47] Aubry M, Finke J, Teissier A, Roche C, Broult J, Paulous S, et al. Seroprevalence of arboviruses among blood donors in French Polynesia, 2011–2013. *Int J Infect Dis*. 2015;41:11–2. doi: 10.1016/j.ijid.2015.10.005 26482390

[48] Aubry M, Teissier A, Huart M, Merceron S, Vanhomwegen J, Roche C, et al. Ross river virus seroprevalence, French Polynesia, 2014–2015. *Emerg Infect Dis*. 2017;23(10):1751. doi: 10.3201/eid2310.170583 28930020 PMC5621548

[49] Aubry M, Teissier A, Huart M, Merceron S, Vanhomwegen J, Mapotoeke M, et al. Seroprevalence of dengue and chikungunya virus antibodies, French Polynesia, 2014–2015. *Emerg Infect Dis*. 2018;24(3):558. doi: 10.3201/eid2403.171149 29460745 PMC5823337

[50] Aubry M, Kama M, Vanhomwegen J, Teissier A, Mariteragi-Helle T, Hue S, et al. Ross River virus antibody prevalence, Fiji Islands, 2013–2015. *Emerg Infect Dis*. 2019;25(4):827. doi: 10.3201/eid2504.180694 30882332 PMC6433005

[51] Darcy AW, Kanda S, Dalipanda T, Joshua C, Shimono T, Lamaningao P, et al. Multiple arboviral infections during a DENV-2 outbreak in Solomon Islands. *Trop Med Health*. 2020;48:1–11. doi: 10.1186/s41182-019-0188-z 32435149 PMC7225641

[52] Gyawali N, Taylor-Robinson AW, Bradbury RS, Pederick W, Faddy HM, Aaskov JG. Neglected Australian Arboviruses Associated With Undifferentiated Febrile Illnesses. *Front Microbiol*. 2019;10(2818). doi: 10.3389/fmicb.2019.02818 31866981 PMC6908948

[53] Duncombe J, Lau C, Weinstein P, Aaskov J, Rourke M, Grant R, et al. Seroprevalence of dengue in American Samoa, 2010. *Emerg Infect Dis*. 2013;19(2):324–6. doi: 10.3201/eid1902.120464 23343610 PMC3559036

[54] Luang-Suarkia D, Ernst T, Alpers MP, Garruto R, Smith D, Imrie A. Serological evidence for transmission of multiple dengue virus serotypes in Papua New Guinea and West Papua prior to 1963. *PLoS Negl Trop Dis*. 2017;11(4):e0005488. Epub 2017/04/25. doi: 10.1371/journal.pntd.0005488 28437465 PMC5426789

[55] Seglenieks Z, Moore BW. Epidemic polyarthritis in South Australia: report of an outbreak in 1971. *Med J Aust*. 1974;2(15):552–6. Epub 1974/10/12. doi: 10.5694/j.1326-5377.1974.tb70993.x 4473695

[56] Mudge PR. A survey of epidemic polyarthritis in the Riverland area, 1976. *Med J Aust*. 1977;1(18):649–51. Epub 1977/04/30. doi: 10.5694/j.1326-5377.1977.tb130990.x 875818

[57] Kanamitsu M, Taniguchi K, Urasawa S, Ogata T, Wada Y, Wada Y, et al. Geographic distribution of arbovirus antibodies in indigenous human populations in the Indo-Australian archipelago. *Am J Trop Med Hyg*. 1979;28(2):351–63. Epub 1979/03/01. doi: 10.4269/ajtmh.1979.28.351 453438

[58] Hii J, Dyke T, Dagoro H, Sanders RC. Health impact assessments of malaria and Ross River virus infection in the Southern Highlands Province of Papua New Guinea. *P N G Med J*. 1997;40(1):14–25. Epub 1997/03/01. 10365566

[59] Allchin L PH, Truman G, Hort K.,. Ross River Virus in Western Sydney: A Serological Survey. N S W *Public Health Bull*. 2003.;14(11–12):224–6. doi: 10.1071/nb03061 14981558

[60] Aubry M, Kama M, Henderson AD, Teissier A, Vanhomwegen J, Mariteragi-Helle T, et al. Low chikungunya virus seroprevalence two years after emergence in Fiji. *Int J Infect Dis*. 2020;90:223–5. Epub 2019/11/07. doi: 10.1016/j.ijid.2019.10.040 31689529 PMC6912130

[61] Maguire T. Do Ross River and dengue viruses pose a threat to New Zealand? *N Z Med J*. 1994;107(989):448–50. . 7970354

[62] O’Neill SL, Ryan P, Turley AP, Wilson G, Retzki K, Iturbe-Ormaetxe I, Dong Y, Kenny N, Paton CJ, Ritchie SA, Brown-Kenyon J, Stanford D, Wittmeier N, Jewell NP, Tanamas SK, Anders KL, Simmons CP. Scaled deployment of Wolbachia to protect the community from dengue and other Aedes transmitted arboviruses. *Gates Open Res*. 2019;2:36. doi: 10.12688/gatesopenres.12844.3 30596205 PMC6305154

[63] Marshall ID WG, Hirsch S. Viruses recovered from mosquitoes and wildlife serum collected in the Murray Valley of Southeastern Australia February 1974 during an epidemic of encephalitis. *Aust J Biol Exp Med Sci*. 1982;60(5):456–70. doi: 10.1038/icb.1982.51 6299258

[64] Partidos CD PJ, Weger J, Borland EM, Powers AM, Seymour R et al. Cross-protective immunity against o’nyong-nyong virus afforded by a novel recombinant chikungunya vaccine. *Vaccine*. 2012;30:4638–43. doi: 10.1016/j.vaccine.2012.04.099 22583812 PMC3372665

[65] Asebe G, Michlmayr D, Mamo G, Abegaz WE, Endale A, Medhin G, et al. Seroprevalence of yellow fever, Chikungunya and Zika virus at a community level in the Gambella Region, South West Ethiopia. *PLoS One*. 2021;16(7):e0253953. doi: 10.1371/journal.pone.0253953 34237098 PMC8266044

[66] Morgan J, Strode C, S-S JE. Climatic and socio-economic factors supporting the co-circulation of dengue, Zika and chikungunya in three different ecosystems in Colombia. *PLoS Negl Trop Dis*. 2021;15(3):e0009259. doi: 10.1371/journal.pntd.0009259 33705409 PMC7987142

[67] Kerkhof K, Falconi-Agapito F, Van Esbroeck M, Talledo M, A KK. Reliable Serological Diagnostic Tests for Arboviruses: Feasible or Utopia? *Trends Microbiol* 2020;28(4):276–92. doi: 10.1016/j.tim.2019.11.005 31864844

