# Supplementary figures and images for: Human Seroprevalence for Dengue, Ross River, and Barmah Forest viruses in Australia and the Pacific: A systematic review spanning seven decades
Source: PLoS Negl Trop Dis. 2022 Apr 29;16(4):e0010314. doi: 10.1371/journal.pntd.0010314 (PMC9094520; doi:10.1371/journal.pntd.0010314)

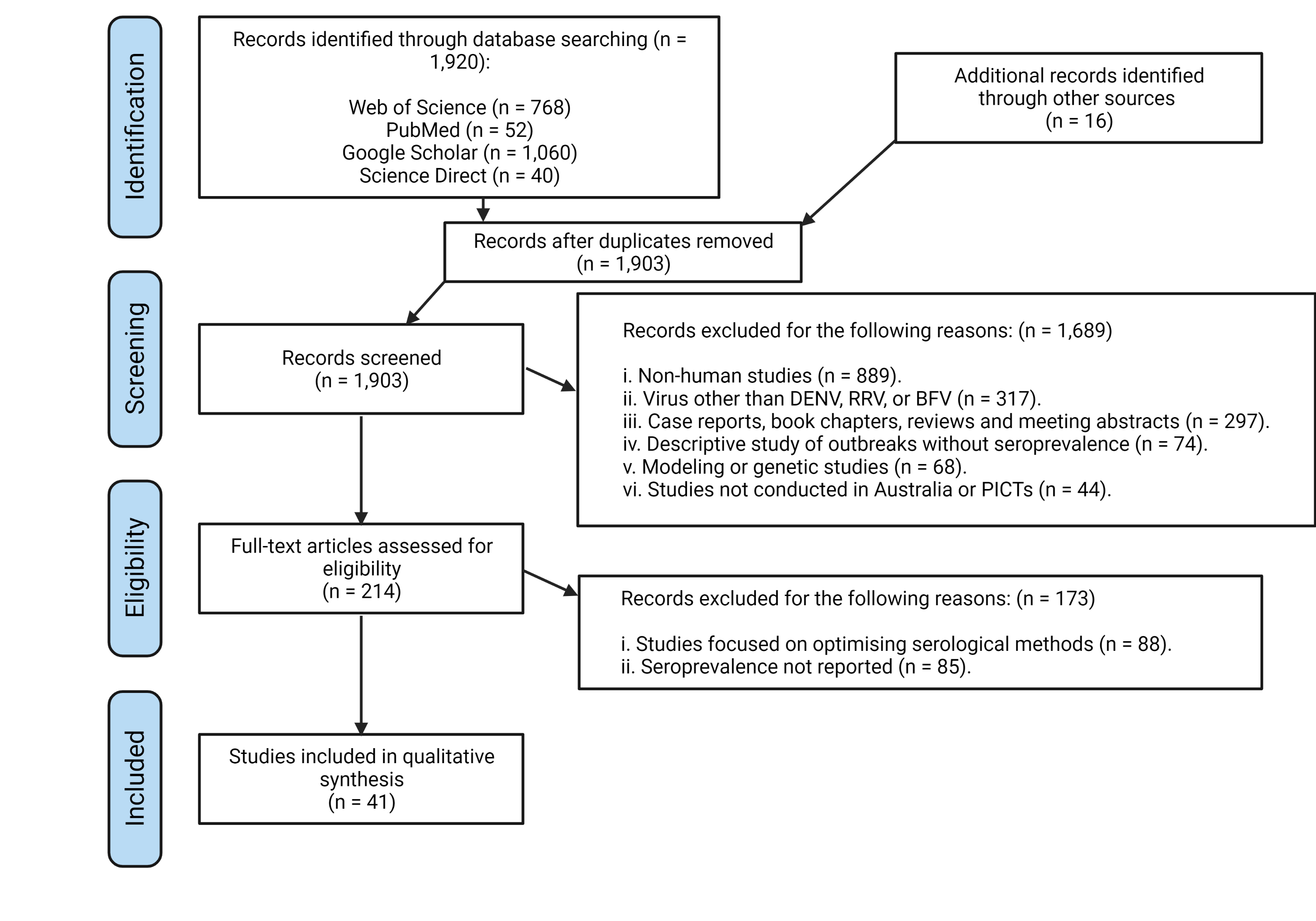

Supplement: S1 Fig — Flow diagram of the systematic search strategy used to identify and include studies into our systematic review of reported DENV, RRV and BFV seroprevalence among humans in Australia and the PICTs. (TIF) [file pntd.0010314.s002.tif]
