# Supplementary material for: Human Seroprevalence for Dengue, Ross River, and Barmah Forest viruses in Australia and the Pacific: A systematic review spanning seven decades
Source: PLoS Negl Trop Dis. 2022 Apr 29;16(4):e0010314. doi: 10.1371/journal.pntd.0010314 (PMC9094520; doi:10.1371/journal.pntd.0010314)
Supplement: S1 Table — (DOCX) [file pntd.0010314.s004.docx]

**S1 Table. Studies reported seroprevalences across age groups**

|  |  |  |  | Age, % (Positive tests/Total tests) | | | | | | | |
| --- | --- | --- | --- | --- | --- | --- | --- | --- | --- | --- | --- |
| Study | **Virus** | **Research area** | **Method** | **0-9 y/o** | **10-19 y/o** | **20-29 y/o** | **30-39 y/o** | **40-49 y/o** | **50-59 y/o** | **60-69 y/o** | **70-79 y/o** |
| Doherty,1966 [1] | RRV | Queensland | HI or NT | 30.97 | 42.95 | 61.95 | | | | | |
| Doherty,1968 [2] | RRV | Near Innisfail | HI | 10.53 | 27.45 | 35.37 | 43.64 | 76.47 | 76.32 (29/38) | | |
| Doherty,1973 (1) [3] | RRV | Northern and eastern Queensland | HI | 0-4 y/o, 26.4 (39/148) | 5-9 y/o, 40.7 (55/135) | 10-14 y/o, 65.2 (92/141) | 15-19 y/o, 57.7 (30/52) | 20-29 y/o, 65 (13/20) | ≥ 40 y/o, 90.9 (10/11) |  |  |
| Doherty,1973 (2) [3] | RRV | Eastern Queensland | HI | 7.05 | 26.11 | 41.06 | 57.98 | 56.63 | 61.4 | 64.71 (44/68) | |
| Liehne,1976 [4] | RRV | Western Australia | HI |  | |  | | | | | |
| Stallman,1976 [5] | RRV | Queensland and Northern Territory | HI | 7.57 | 11.11 |  |  |  |  |  |  |
| Kanamitsu,1979 [6] | RRV | Queensland | HI | 5 | 22 | 30 | 48 | 36 | 43 | | |
| Aaskov,1981[7] | RRV | Western Viti Lev | HI | 4.33 | | 42.31 | | | | | |
| Fraser,1986 [8] | RRV | Echuca | HI | 5-24 y/o, 4.21 (9/214) | 25-44 y/o, 7.98 (17/213) | 45-64 y/o, 16.90 (36/213) | ≥ 65 y/o, 28.28 (28/99) |  |  |  |  |
| Hawkes,1993* [9] | RRV | New South Wales | HI | 17.31 | 19.9 | 28.71 | 33.14 | 40-49 y/o, 38.24 (169/442) | 45.75 | 44.55 | |
| Weinstein,1994 ** [10] | RRV | South Australia | ELISA(IgG) | NA |  |  |  |  |  |  |  |
| Hii, 1997** [11] | RRV | Nogolitogo, Papua New Guinea | ELISA(IgG) | NA |  |  |  |  |  |  |  |
| Dodsley,2001** [12] | RRV | Western Australia | HI | NA |  |  |  |  |  |  |  |
| Faddy,2015 (1-2) [13] | RRV | Queensland and Murray Valley | ELISA(IgM) | ≤ 24 y/o, 0.54 (4/747) | 25-34 y/o, 0.71 (5/704) | 35-44 y/o, 0.53 (4/755) | 45-54 y/o, 1.43 (19/1332) | 55-64 y/o, 0.84 (10/1184) | ≥ 65 y/o, 1.02 (4/392) |  |  |
| Aubry,2019 (1) [14] | RRV | Fiji | MIA (IgG) | 23.03 | 45.21 | 49.57 | 58.63 | | | | |
| Aubry,2019 (2) [14] | RRV | Fiji | MIA (IgG) | 22.35 | 30.3 | 44.74 | 47.22 | | | | |
| Phillips,1990 (1) [15] | RRV | Queensland | HI | 6.78 | 19.07 | 27.8 | 31.3 | 35.34 | 41.33 | 45.76 | 51.92 |
| Phillips,1990 (2) [15] | BFV | Queensland | HI | 0 | 1.69 | 3.81 | 6.1 | 8.38 | 10.67 | 11.86 | 17.31 |
| McBride,1998 (2)* [16] | DENV | Charters Towers | NT and (HI or ELISA) | 8 | | | 22 | 30 | 51-64 y/o, 52.00 |  | 65-88 y/o, 60.00 |
| Faddy,2013 (2) [17] | DENV | Queensland | ELISA(IgG) | 3.17 | | | | 9.43 | | 24.27 | |
| Faddy,2013 (3) [17] | DENV | Melbourne | ELISA(IgG) | 7.28 | | | | 7.44 | | 4.4 | |
| Darcy,2020 [18] | DENV | Honiara and Gizo | ELISA(IgG) | 1-5 y/o, 61.5 (16/26) | 6-10 y/o, 75.0 (18/24) | 11-15 y/o, 78.8 (26/33) | 16-20 y/o, 95.0 (19/20) | - | - | - | - |
| Hawkes,1987 [19] | BFV | New South Wales | ELISA(IgG) and HI | 1.58 | | 1.51 | | 2.82 | | | |
| Faddy,2015 (10-11) [13] | BFV | Queensland and Murray Valley | ELISA(IgM) | ≤ 24 y/o, 1.57 (11/699) | 25-34 y/o, 1.61 (11/683) | 35-44 y/o, 0.67 (5/744) | 45-54 y/o, 1.41 (18/1281) | 55-64 y/o, 0.67 (8/1201) | ≥ 65 y/o, 1.88 (8/425) | - | - |

Abbreviations: DENV = Dengue virus, RRV = Ross River virus, BFV = Barmah Forest virus, NA = Not Available, HI = Hemagglutination Inhibition test, NT = Neutralisation test, ELISA = Enzyme-Linked Immunosorbent Assay. ^*^The numbers were estimated from rates or the rates were estimated from figures. ^**^Unable to extract the exact seroprevalence in age groups from the figures. ***Age range were estimated according to the age groups.

**References**

1. Doherty RL, Gorman B, Whitehead R, Carley J. Studies of Arthropod‐borne Virus Infections in Queensland: V. Survey of Antibodies to Group A Arboviruses in Man and other Animals. Australian Journal of Experimental Biology and Medical Science. 1966;44(4):365-78.

2. Doherty R, Standfast H, Wetters E, Whitehead R, Barrow G, Gorman B. Virus isolation and serological studies of arthropodborne virus infections in a high rainfall area of north queensland. Transactions of the Royal Society of Tropical Medicine and Hygiene. 1968;62(6):862-7.

3. Doherty RL. Surveys of haemagglutination-inhibiting antibody to arboviruses in aborigines and other population groups in northern and eastern Australia, 1966–1971. Transactions of the Royal Society of Tropical Medicine and Hygiene. 1973;67(2):197-205.

4. Liehne C, Stanley N, Alpers M, Paul S, Liehne P, Chan K. Ord River arboviruses—serological epidemiology. Australian Journal of Experimental Biology and Medical Science. 1976;54(5):505-12.

5. Stallman N, Wiemers M, Bourke A. Serology in diagnosis and surveys of man. Arbovirus Res Aust. 1976;1:46-64.

6. Kanamitsu M, Taniguchi K, Urasawa S, Ogata T, Wada Y, Wada Y, et al. Geographic distribution of arbovirus antibodies in indigenous human populations in the Indo-Australian archipelago. Am J Trop Med Hyg. 1979;28(2):351-63. Epub 1979/03/01. doi: 10.4269/ajtmh.1979.28.351. PubMed PMID: 453438.

7. Aaskov J, Mataika J, Lawrence GW, Rabukawaga V, Tucker MM, Miles JA *et al*.,. An epidemic of Ross River virus infection in Fiji. Am J Trop Med Hyg. 1981;30(5):1053 - 9. doi: 10.4269/ajtmh.1981.30.1053.; PubMed Central PMCID: PMCPMID: 7278766.

8. Fraser J, Christie D, Gust I, White J, Leach R, Macaulay E, et al. Arbovirus infection in a murray valley community. Australian and New Zealand journal of medicine. 1986;16(1):52-7.

9. Hawkes RA, Pamplin J, Nairn HM, Boughton CR. Arbovirus infections of humans in high‐risk areas of south‐eastern Australia: a continuing study. Medical journal of Australia. 1993;159(3):159-62.

10. Weinstein P, Cameron S, Worswick D, McIntyre A. Human sentinels for arbovirus surveillance and regional risk classification in South Australia. Medical journal of Australia. 1994;160(8):494-9.

11. Hii J, Dyke T, Dagoro H, Sanders RC. Health impact assessments of malaria and Ross River virus infection in the Southern Highlands Province of Papua New Guinea. P N G Med J. 1997;40(1):14-25. Epub 1997/03/01. PubMed PMID: 10365566.

12. Dodsley N, Broom A, Smith D, Plant A, Lindsay M. Ross River virus: determining the prevalence in the south west of Western Australia. Arbovirus Research in Australia. 2001;8:122-5.

13. Faddy H, Dunford M, Seed C, Olds A, Harley D, Dean M, et al. Seroprevalence of antibodies to Ross River and Barmah Forest viruses: Possible implications for blood transfusion safety after extreme weather events. EcoHealth. 2015;12(2):347-53.

14. Aubry M, Kama M, Vanhomwegen J, Teissier A, Mariteragi-Helle T, Hue S, et al. Ross River virus antibody prevalence, Fiji Islands, 2013–2015. Emerging infectious diseases. 2019;25(4):827.

15. Phillips DA, Murray JR, Wiemers MA, Aaskov JG. Clinical and subclinical Barmah Forest virus infection in Queensland. Medical Journal of Australia. 1990;152(9):463-6.

16. McBride W, Mullner H, LaBrooy JT, Wronski I. The 1993 dengue 2 epidemic in North Queensland: a serosurvey and comparison of hemagglutination inhibition with an ELISA. The American journal of tropical medicine and hygiene. 1998;59(3):457-61.

17. Faddy HM, Seed CR, Fryk JJ, Hyland CA, Ritchie SA, Taylor CT, et al. Implications of dengue outbreaks for blood supply, Australia. Emerging infectious diseases. 2013;19(5):787.

18. Darcy AW, Kanda S, Dalipanda T, Joshua C, Shimono T, Lamaningao P, et al. Multiple arboviral infections during a DENV-2 outbreak in Solomon Islands. Tropical medicine and health. 2020;48:1-11.

19. Hawkes RA, Nairn HM, Myrick BM, Ramsay LG. Barmah Forest virus infections in humans in New South Wales. Medical journal of Australia. 1987;146(11):569-73.
