# Supplementary material for: Human Seroprevalence for Dengue, Ross River, and Barmah Forest viruses in Australia and the Pacific: A systematic review spanning seven decades
Source: PLoS Negl Trop Dis. 2022 Apr 29;16(4):e0010314. doi: 10.1371/journal.pntd.0010314 (PMC9094520; doi:10.1371/journal.pntd.0010314)
Supplement: S2 Table — (DOCX) [file pntd.0010314.s005.docx]

**S2 Table. Studies reported seroprevalences across gender groups**

| Study | Virus | Research area^*^ | Method | Gender, % (Positive tests/Total tests) | |
| --- | --- | --- | --- | --- | --- |
|  |  |  |  | **Male** | **Female** |
| McBride,1998 (1)^**^ [1] | DENV | Charters Towers | ELISA(IgG) | 63.0 | 61.0 |
| McBride,1998 (2)^**^ [1] | DENV | Charters Towers | NT and (HI or ELISA) | 38.0 | 41.0 |
| Darcy,2020 [2] | DENV | Honiara and Gizo | ELISA(IgG) | 77.1 (64/83) | 88.6 (93/105) |
| Doherty,1968 [3] | RRV | Near Innisfail | HI | 46.2 (114/247) | 37.8 (14/37) |
| Doherty,1973 (2) [4] | RRV | Eastern Queensland | HI | 45.0 (327/727) | 39.0 (287/736) |
| Fraser,1986 [5] | RRV | Echuca | HI | 12.1 (43/355) | 12.2 (47/384) |
| Hawkes,1993^**^ [6] | RRV | New South Wales | HI | 32.6 (389/1193) | 28.8 (455/1578) |
| Faddy,2015 (1-2) [7] | RRV | Queensland and Murray Valley | ELISA(IgM) | 0.9 (25/2680) | 0.9 (21/2434) |
| Aubry,2019 (1) [8] | RRV | Fiji | ^***^ | 47.2 (167/354) | 46.1 (195/423) |
| Aubry,2019 (2) [8] | RRV | Fiji | ^***^ | 35.7 (51/143) | 38.4 (73/190) |
| Phillips,1990 (1) [9] | RRV | Queensland | HI | 34.2 (358/1046) | 28.8 (278/964) |
| Hawkes,1987 [10] | BFV | New South Wales | ELISA(IgG) and HI | 2.5 (45/1785) | 1.4 (25/1755) |
| Phillips,1990 (2) [9] | BFV | Queensland | HI | 7.8 (82/1046) | 5.0 (48/964) |
| Faddy,2015 (10-11) [7] | BFV | Queensland and Murray Valley | ELISA(IgM) | 1.3 (34/2637) | 1.1 (27/2396) |

Abbreviations: DENV = Dengue virus, RRV = Ross River virus, BFV = Barmah Forest virus, HI = Hemagglutination Inhibition test, NT = Neutralisation test, ELISA = Enzyme-Linked Immunosorbent Assay. ^*^If the research area includes more than one area, a larger area contains these areas was used. ^**^The number of positive tests were estimated from rates reported in papers. ^***^RRV IgG using a recombinant antigen-based microsphere immunoassay (MIA).

**References**

1. McBride W, Mullner H, LaBrooy JT, Wronski I. The 1993 dengue 2 epidemic in North Queensland: a serosurvey and comparison of hemagglutination inhibition with an ELISA. The American journal of tropical medicine and hygiene. 1998;59(3):457-61.

2. Darcy AW, Kanda S, Dalipanda T, Joshua C, Shimono T, Lamaningao P, et al. Multiple arboviral infections during a DENV-2 outbreak in Solomon Islands. Tropical medicine and health. 2020;48:1-11.

3. Doherty R, Standfast H, Wetters E, Whitehead R, Barrow G, Gorman B. Virus isolation and serological studies of arthropodborne virus infections in a high rainfall area of north queensland. Transactions of the Royal Society of Tropical Medicine and Hygiene. 1968;62(6):862-7.

4. Doherty RL. Surveys of haemagglutination-inhibiting antibody to arboviruses in aborigines and other population groups in northern and eastern Australia, 1966–1971. Transactions of the Royal Society of Tropical Medicine and Hygiene. 1973;67(2):197-205.

5. Fraser J, Christie D, Gust I, White J, Leach R, Macaulay E, et al. Arbovirus infection in a murray valley community. Australian and New Zealand journal of medicine. 1986;16(1):52-7.

6. Hawkes RA, Pamplin J, Nairn HM, Boughton CR. Arbovirus infections of humans in high‐risk areas of south‐eastern Australia: a continuing study. Medical journal of Australia. 1993;159(3):159-62.

7. Faddy H, Dunford M, Seed C, Olds A, Harley D, Dean M, et al. Seroprevalence of antibodies to Ross River and Barmah Forest viruses: Possible implications for blood transfusion safety after extreme weather events. EcoHealth. 2015;12(2):347-53.

8. Aubry M, Kama M, Vanhomwegen J, Teissier A, Mariteragi-Helle T, Hue S, et al. Ross River virus antibody prevalence, Fiji Islands, 2013–2015. Emerging infectious diseases. 2019;25(4):827.

9. Phillips DA, Murray JR, Wiemers MA, Aaskov JG. Clinical and subclinical Barmah Forest virus infection in Queensland. Medical Journal of Australia. 1990;152(9):463-6.

10. Hawkes RA, Nairn HM, Myrick BM, Ramsay LG. Barmah Forest virus infections in humans in New South Wales. Medical journal of Australia. 1987;146(11):569-73.
